# The Cut-off Value of Physical Activity for Undergoing Total Knee Arthroplasty in Patients with Knee Osteoarthritis

**DOI:** 10.3390/healthcare9081063

**Published:** 2021-08-19

**Authors:** Takuya Okamoto, Tatsunori Ikemoto, Hirofumi Miyagawa, Tomohiro Ishida, Machiko Akao, Takuya Takata, Kyosuke Kobayakawa, Yuki Yamanashi, Masayuki Inoue, Yuichiro Nakaso, Takahiro Ushida, Masataka Deie

**Affiliations:** 1 Institute of Physical Fitness, Sports Medicine and Rehabilitation, Aichi Medical University, Nagakute 480-1131, Japan; ptmiya47@gmail.com (H.M.); masayukiino17@gmail.com (M.I.); nakaso.y@gmail.com (Y.N.); ushidat@aichi-med-u.ac.jp (T.U.); 2 Department of Rehabilitation, Aichi Medical University Hospital, Nagakute 480-1131, Japan; ishida.tomohiro.758@mail.aichi-med-u.ac.jp; 3 Department of Orthopaedic Surgery, Aichi Medical University, Nagakute 480-1131, Japan; tatsunon31@gmail.com (T.I.); akao@aichi-med-u.ac.jp (M.A.); takuyasdagger123@gmail.com (T.T.); kobayakawa.kyousuke.634@mail.aichi-med-u.ac.jp (K.K.); yuuki.6120.22@gmail.com (Y.Y.); snm3@aichi-med-u.ac.jp (M.D.); 4 Multidisciplinary Pain Center, Aichi Medical University, Nagakute 480-1131, Japan

**Keywords:** knee osteoarthritis, total knee arthroplasty, physical activity, moderate-to-vigorous physical activity

## Abstract

Background: We aimed to determine a cut-off value for physical activity (PA), measured using an accelerometer, between patients with knee osteoarthritis (OA) who decided to undergo total knee arthroplasty (TKA) and those who continued conservative treatment. Methods: Forty-two participants were assigned to either a TKA group or a non-TKA group (21 per group). They were instructed to wear an accelerometer throughout the day. Average daily steps (steps/day), average daily time of light PA (LPA) (min/day), and average daily time of moderate-to-vigorous PA (MVPA) (min/day) were measured for seven days. Variables between the two groups were compared using univariate analyses, and then a stepwise logistic regression was conducted to determine which variables best correlated with undergoing TKA. The PA cut-offs were analysed using the receiver operating characteristic curve. Results: Pain severity (*p* = 0.002), KL grade (*p* = 0.001), and MVPA (*p* = 0.012) differed significantly between the groups. The most useful cut-off value was 5.84 (min/day) for MVPA (AUC = 0.773), although only pain severity and KL grade were found to be significant contributors to undergoing TKA. Conclusions: Our results revealed there was a significant decrease in PA levels (MVPA cut-off, 5–6 min/day) in the TKA group compared with the non-TKA group.

## 1. Introduction

Knee osteoarthritis (OA) is a degenerative disease that causes knee pain and disability among older people worldwide [1]. In a Japanese cohort study, 82.5% of people aged ≥60 years had knee OA [2]. Many guidelines have reported that treatment strategies for symptomatic knee OA include stepwise treatment options such as voluntary physical exercise, weight control education, physiotherapy, use of analgesic drugs such as non-steroidal anti-inflammatory drugs and acetaminophen, and intra-articular injection of steroids and hyaluronic acids [3,4]. Total knee arthroplasty (TKA) is often chosen as the final option for patients with knee OA when conservative treatments fail, and TKA has been reported to result in good outcomes, including pain relief and improved walking ability among these populations.

Several guidelines have reported that the indication criteria for TKA includes pain, function, and radiological severity of knee OA [5,6,7]. The Kellgren–Lawrence (KL) grade is the most commonly used X-ray-based grading criteria for assessing the severity of knee OA [8]; KL grade III or higher is commonly considered an indication for TKA [5,6,7]. However, it is unclear how clinicians estimate treatment failures, because pain is a subjective experience, and it is changeable over time [9]. Furthermore, it is widely known that radiographic disease severity is not always associated with symptom severity [10].

The therapeutic goals in knee OA include reducing pain and improving functional disability. As levels of “physical activity (PA)” are associated with functional disability [11], decreased PA in patients with knee OA is also considered an indicator for TKA [12]; however, there has been no information on a PA cut-off value indicative of TKA surgery.

PA measures often include objectively measured moderate-to-vigorous PA (MVPA), light PA (LPA), and steps [13]. In recent years, MVPA, which refers to activities corresponding to more than three metabolic equivalents (METs) [14], has been widely used to assess PA. It has also been reported that a lower MVPA level is associated with higher all-cause mortality rates [15] as well as quality of life [16]. In 2008, the American PA Guidelines recommended a minimum of 10 min of MVPA per day for substantial health benefits for older people [17]. According to a World Health Organization (WHO) statement, a 150 min MVPA exercise is recommended per week for older people; however, it was reported that nearly half (46.3%) of the older Japanese population living in rural communities did not perform ≥150 min/week of MVPA [18]. Matsunaga et al. also reported that the mean (±standard deviation) duration of MVPA in patients with knee OA before TKA was only 2.9 (±4.3) min/day [19]. Therefore, we speculated that there is a cut-off value for MVPA in patients who undergo TKA.

This study aimed to determine the cut-off value for MVPA, which was measured using an accelerometer, between patients with knee OA who underwent TKA (TKA group) and those who continued conservative treatment (non-TKA group). We hypothesized that MVPA levels in patients with knee OA were significantly lower in the TKA group than in the non-TKA group.

## 2. Materials and Methods

### 2.1. Study Design and Ethics

In this cross-sectional study, we applied the Strengthening the Reporting of Observational Studies in Epidemiology statement for cross-sectional investigation [20]. This study was approved by the Ethics Committee of our institution (ethic No. 2020-175).

### 2.2. Sample Size

The number of participants was determined by a sample size estimation using EZR (version 1.42, Saitama Medical Center, Jichi Medical University, Saitama, Japan), which is a graphical user interface for the R software (The R Foundation for Statistical Computing, Vienna, Austria) [21]. Since we speculated a possible cut-off value for MVPA between the TKA and non-TKA groups, the receiver operating characteristic (ROC) curve was used to detect this value. The area under the ROC curve (AUC) is an effective method for summarizing the overall diagnostic accuracy of a test. In general, an AUC of 0.5 suggests no discrimination, while 0.7–0.8 is considered acceptable [22]. In the preliminary 10 cases, we postulated that there was a 74% chance that the model could distinguish between the two groups. For an AUC of 0.74, a two-tailed α level of 0.05, a power (1-β) of 0.8, and a 1:1 allocation, the minimum number of subjects was estimated to be 21 for each group. Therefore, we recruited 42 participants (21 per group) in the order of their visits to our clinic, in accordance with per-protocol analysis.

### 2.3. Enrolment

We enrolled patients with symptomatic knee OA at our orthopedic clinic between October 2019 and September 2020. The inclusion criteria for this study were as follows: (1) knee pain, (2) age ≥ 60 years, (3) >1 year of treatment history for knee OA, and (4) presence of radiographic knee OA with KL grade II or higher [8]. Exclusion criteria were as follows: (1) presence or history of major neurological disorders such as stroke or Parkinson’s disease, (2) history of contralateral knee operation, (3) presence of hip/ankle OA or history of total hip/ankle arthroplasty, (4) under treatment for lumbar canal stenosis, (5) ongoing traumatic disease, (6) malignant disease, (7) autoimmune disease such as rheumatoid arthritis, and (8) dementia. Investigators asked eligible patients to participate in this study; patients participated in this study voluntarily, and they were free to decline or discontinue their participation at any time. After being informed of the purpose and protocols of the study, all participants provided written informed consent.

### 2.4. Patient Characteristics and Assessment of Disease Severity

First, we investigated the participants’ age, sex, and body mass index (BMI). BMI was calculated from weight and height measurements using the formula BMI = weight in kilograms (kg) divided by height in meters squared (m^2^). Radiographic examination of the affected knee from the posterior–anterior view in the fixed standing position was performed by radiological technicians. All radiographs were assessed by at least two orthopedic physicians according to the KL grading system [8]. We used the following 5-point verbal rating scale to assess the severity of knee pain: 0: no pain; 1: mild pain; 2: moderate pain; 3: considerable pain; 4: extreme pain [23].

### 2.5. Decision Undergoing TKA

Standard conservative treatments such as weight control education, exercise, use of oral and topical analgesic agents, and/or intra-articular injections were performed for at least one year for eligible patients with knee OA. When standard conservative treatments were not satisfactory for those patients, TKA was suggested by their attending physicians as a final treatment option in accordance with the following criteria: (1) age ≥ 70 years, (2) KL grade III or IV, and (3) osteoarthritic changes in at least two parts out of the following three compartments: a patellofemoral joint and both condyles of the tibiofemoral joint. The patients were sufficiently informed about the general risks and benefits of TKA surgery. However, even when the patient did not meet all of the above criteria, TKA was chosen when patients complained of severe symptoms and disability and had an enthusiastic desire for TKA surgery. The final decision to undergo TKA depended on the patient’s preference.

### 2.6. Measurement of PA

The PA of the participants was measured using an accelerometer. All participants were instructed to wear an accelerometer (Lifecorder^®^, Suzuken Co. Ltd., Nagoya, Japan) at the waist level throughout the day, except during water-related activities (e.g., bathing) because of its non-waterproof function. Lifecorder is a validated device with good reliability [24], and several studies have used this device for measuring PA in patients with knee OA [19,25].

PA measurement periods were the last 14 days before admission for the TKA group and 14 consecutive days for the non-TKA group. The initial and last days and days when PA was not recorded for more than three consecutive hours in a day were excluded from the analysis of PA measurements. Finally, the average PA for 7 days for each participant was used for analysis. Participants who did not have PA data for at least 7 days or those whose accelerometer did not work properly were excluded due to incomplete data, in accordance with the method reported previously [26].

The acceleration signal was filtered using an analogue bandpass filter and digitized. A maximum pulse rate of over 4 s was considered as the acceleration value, and the activities were categorized into 11 levels (0.0, 0.5, and 1.0–9.0) based on the pattern of the accelerometric signal. Software (Liferiser Coach 05^®^, Suzuken Co. Ltd., Nagoya, Japan) was used to retrieve the PA data from the accelerometer. PA was automatically recorded as step counts and divided into different intensity levels: levels 0, 0.5, and 1.0–9.0. These activity levels were also automatically transformed to corresponding estimated METs, as follows; level 1.0:1.8 METs, level 2.0:2.3 METs, level 3.0:2.9 METs, level 4.0:3.6 METs, level 5.0:4.3 METs, level 6.0:5.2 METs, level 7.0:6.1 METs, level 8.0:7.1 METs, and level 9.0:>8.3 METs. The working principle of this device has been described previously [27]. In accordance with this principle, LPA was defined as activities between level 1.0 and level 3.0, and MVPA was defined as activities that were level 4.0 or above [27].

Finally, the following indicators were adopted as parameters of PA in the present study: average daily steps (steps/day), average daily time of LPA (min/day), and average daily time of MVPA (min/day).

### 2.7. Allocation

**Figure 1:**
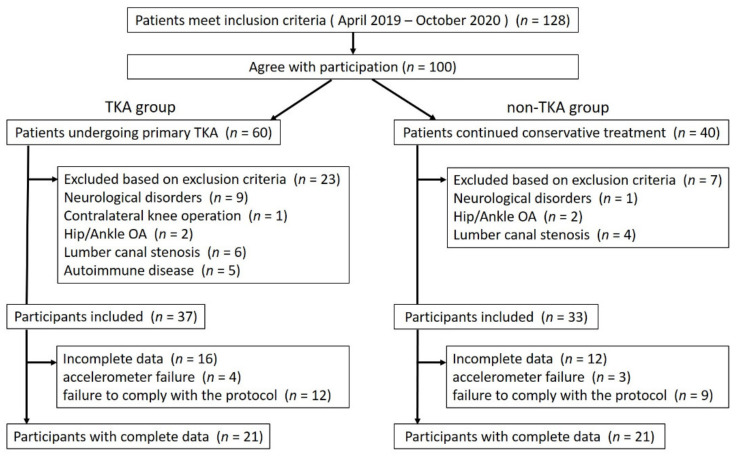
Flowchart of patient selection in this study. Abbreviations: TKA, total knee arthroplasty; OA, osteoarthritis.

One hundred and twenty-eight patients with knee OA met the inclusion criteria. One hundred patients agreed to participate; of them, 60 patients underwent TKA (TKA group), while 40 patients continued conservative treatment (non-TKA group). Twenty-three patients in the TKA group and 7 patients in the non-TKA group were excluded from the study, and then 37 patients and 33 patients in each group, respectively, were asked to participate in this study. In the non-TKA group, TKA surgery was not performed within the study period and for at least 6 months after this investigation. Sixteen (43.2%) of the 37 patients in the TKA group and 12 (36.4%) of the 33 patients in the non-TKA group were excluded due to incomplete data. Finally, 42 participants were enrolled in this study (TKA group, *n* = 21; non-TKA group: *n* = 21) (Figure 1).

### 2.8. Statistical Analyses

Continuous variables were represented as means and standard deviations, or medians and interquartile ranges, in accordance with data distributions, while categorical variables were represented as the numbers and percentages of patients. The following analyses were performed using EZR^®^ [21] and SPSS software (version 26, SPSS Inc., Chicago, IL, USA). All results were considered statistically significant at *p* < 0.05.

#### 2.8.1. Comparison of Parameters between the Groups

Variables between the two groups were compared using the Chi-square test, Student’s *t*-test, or Mann–Whitney *U* test for demographic data, disease severity, and PA, according to appropriate fitting models. Then, linear mixed models were used to identify differences in PA between the two groups, correcting for potential confounders: age, sex, BMI, and KL grade.

#### 2.8.2. Cut-off Value Analysis

An ROC curve analysis was performed for PA parameters, which showed a statistically significant difference between the two groups, and the AUC was calculated to evaluate the diagnostic value.

#### 2.8.3. PA-Related Predictors for Undergoing TKA

After comparing PA-related variables between the two groups, a stepwise logistic regression was conducted to determine whether the level of PA was a significant contributor toward undergoing TKA, even after adjusting other variables, and to determine which variables best correlated with undergoing TKA.

## 3. Results

### 3.1. Characteristics of the Participants

The participants included 9 men (21.4%) and 33 women (78.6%). The average age and BMI values were 75.05 years and 25.29 kg/m², respectively. The number of participants according to knee OA disease severity was 6 (14.3%) with KL grade II, 12 (28.6%) with KL grade III, and 24 (57.1%) with KL grade IV (Table 1).

### 3.2. Comparison of Variables between the TKA Group and the Non-TKA Group

Although there were no significant differences in demographic data (age, sex ratio, and BMI) between the two groups, the severity of knee OA was greater in the TKA group than in the non-TKA group. As expected, pain severity was greater in the TKA group than in the non-TKA group (Table 1).

**Figure 2:**
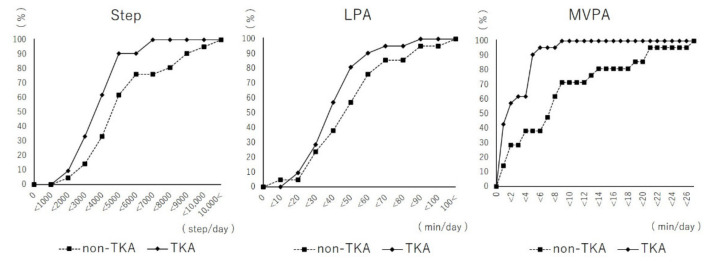
Differences in physical activity levels between the TKA group and the non-TKA group. Lines show an accumulation plot according to the activity levels of the two groups. Abbreviations: LPA, light physical activity; MVPA, moderate-to-vigorous physical activity.

Regarding PA, there was a significant difference in MVPA after controlling for confounders, but not in the number of steps and LPA, between the two groups (Table 1, Figure 2).

### 3.3. Cut-off Value

**Figure 3:**
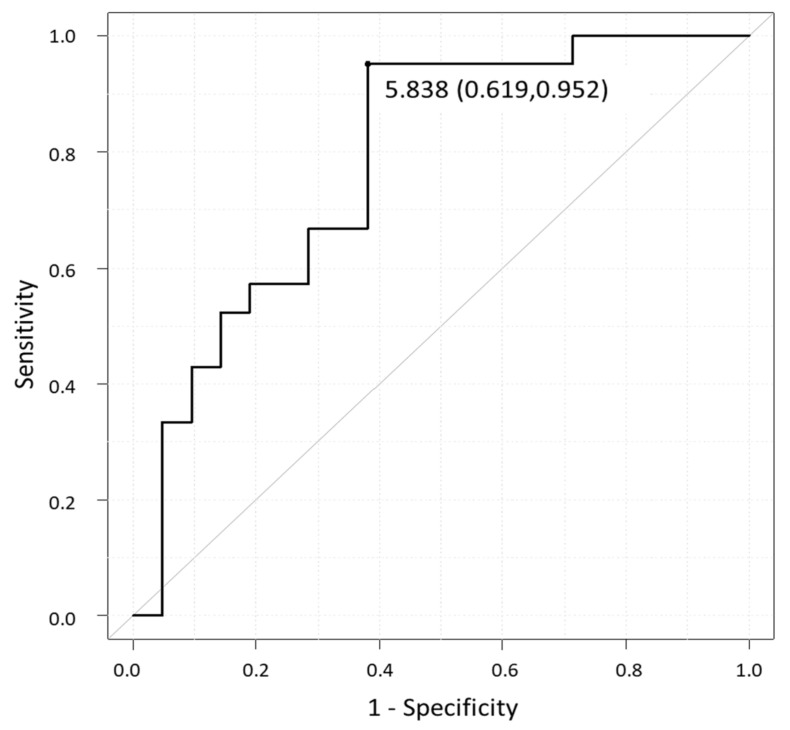
Receiver operating characteristic curve for MVPA. Cut-off value (specificity, sensitivity): 5.838 min/day (0.619, 0.952), AUC (95% CI): 0.773 (0.626–0.921). Abbreviations: MVPA, moderate-to-vigorous physical activity; AUC, area under the curve; CI, confidence interval.

The ROC curve (Figure 3) analysis for MVPA between the two groups showed that the most useful cut-off was 5.84 min/day for MVPA, which had a sensitivity of 61.9%, a specificity of 95.2%, and an AUC of 0.773 (95% confidence interval (CI); 0.626, 0.921).

### 3.4. Predictors for Undergoing TKA

As we found that the KL grade, pain severity, step, and MVPA were potential predictors (*p* < 0.1) of TKA surgery, these four variables were used as independent variables in a stepwise logistic regression model. As a result, we found that significant contributors to undergoing TKA were KL grade (OR = 3.97, *p* = 0.049) and pain severity (OR = 2.75, *p* = 0.019) (Table 2).

## 4. Discussion

This study investigated the cut-off value for decreased PA levels in patients with knee OA who underwent TKA and those who continued conservative treatment. Although the results revealed that the PA cut-off value predictive of TKA surgery was determined by the level of MVPA, with a value of 5.84 (min/day), only the pain severity and KL grade were found to be significant contributors to undergoing TKA. To the best of our knowledge, this is the first report to provide a specific cut-off value for decreased PA levels in patients with knee OA who decided to undergo TKA surgery, even though a direct relationship between a decrease in PA levels and TKA treatment remains unclear.

In this study, pain severity and KL grade were significant variables distinguishing between the two groups, suggesting that pain severity, followed by KL grade, was the determinant for TKA surgery among this study sample. As pain, function, and radiological disease severity are three main indicators for TKA surgery, the TKA group in this study was considered to reflect the general TKA decision process.

However, previous studies often report inconsistent relationships between pain severity and KL grade among patients with knee OA [10]; moreover, it is difficult to define functional disability in patients with knee OA. For example, the terms “pain” and “pain-related disability” are different, especially in patients with chronic pain [28]. Improving physical functioning is one of the key clinical outcomes in patients with intractable chronic pain [29]. Miyagawa et al. reported that pain-related disability significantly improved as a result of a one-year supervised exercise program in patients with advanced knee OA [30]. Therefore, it remains unclear how to estimate functional decline in patients with knee OA undergoing conservative treatment. In contrast, one study reported that the activity level after TKA appeared to be affected by PA level before surgery rather than by the treatment itself [31]. Another study reported that lower levels of preoperative physical function were associated with lower functional recovery after TKA [32].

In this study, the mean MVPA time in patients with knee OA before TKA was only 2.5 (± 2.3) min/day, which is consistent with the result reported by Matsunaga et al. [19]. In other words, patients who decided to undergo TKA surgery in this study had decreased their activity level to approximately 10% of their ideal activity level (21 min/day), as recommended by WHO. On the other hand, Lutzner et al. reported that only 16.5% of patients undergoing TKA met these recommendation levels [33]. Although Matsunaga et al. reported that the preoperative MVPA in patients undergoing TKA significantly improved six months after TKA [19], the improved activity level (mean MVPA: 6.0 min/day) in the subjects was only 30% of the ideal activity level.

We understand that pain is an important factor in the decision to undergo TKA. However, if the severity of pain is the main factor in the decision for a patient with knee OA, the patient may not be aware of the functional decline during longstanding pain aggravation. Given the inadequate functional recovery after TKA, a higher preoperative physical function is required for better postoperative outcomes. Thus, an MVPA value of 5–6 min/day, which is twice the mean MVPA obtained from the TKA group in this study, may serve as an indicator of TKA eligibility to maintain the health status of patients with knee OA after TKA surgery.

This study has some limitations. First, although the sample size was statistically adequate based on our calculations, a larger sample size would be preferred. Additionally, recruitment of the participants from only a single institute in this study may not reflect all characteristics of patients with knee OA, because lifestyle activities of individuals may differ according to their geographic region (i.e., urban or rural). Thus, a multicenter study that includes different geographic regions is required. Second, we were unable to collect data for some cases that met the eligibility criteria because the accelerometer wear time was inadequate during the target period. The compliance issue of the participants might have influenced the results of this study. Third, although other comorbidities aside from major musculoskeletal disorders and medications use were not considered in this analysis, these might affect the results [34,35]. Fourth, treatment strategies were not always consistent for all subjects because treatment selections partly depend on patient preference. Finally, the final decision to undergo TKA surgery depends on the patient’s will. Communication between clinicians and patients may influence these decisions [36]. Therefore, knowledge of best practices may change the cut-off values. A longitudinal study is required to establish a better outcome strategy for patients with knee OA.

## 5. Conclusions

This study showed that there was a significant decrease in the PA levels of patients in the TKA group compared with those in the non-TKA group. It also provided a provisional cut-off value for decreased PA levels; less than 5–6 min/day of MVPA for TKA treatment among older patients with knee OA. However, a longitudinal study is required to establish a better outcome strategy for patients with knee OA.

## Figures and Tables

**Table 1:** Comparison of variable parameters between the TKA and non-TKA groups.

Variables		TKA (*n* = 21)			non-TKA (*n* = 21)			*p*-Value	
		Mean ± SD	Median (IQR)	*p*-Value S–W Test ^#^	Mean ± SD	Median (IQR)	*p*-Value S–W Test ^#^	Non-Adjusted	Adjusted
Epidemiologic	Age (years)	75.86 ± 5.52	75 (71, 79)	0.442	74.24 ± 6.38	74 (70, 78)	0.872	0.384	—
	Sex (m:f)	6:15		—	3:18		—	0.259	—
	BMI (kg/m^2^)	25.28 ± 4.85	23.74 (21.63, 26.92)	0.002 **	25.30 ± 2.88	25.0 (23.3, 26.4)	0.154	0.435	—
	KL grade (II:III:IV)	1:2:18		—	5:10:6		—	0.001 **	—
PA	Step (step/day)	3681.42 ± 1437.10	3614.71 (2906.71, 4631.00)	0.513	5109.37 ± 2449.51	4331.00 (3678.57, 5855.86)	0.109	0.028 *	0.537
	LPA (min/day)	39.86 ± 15.93	36.42 (29.06, 48.77)	0.540	47.86 ± 23.83	45.19 (30.37, 59.11)	0.275	0.208	0.698
	MVPA (min/day)	2.47 ± 2.32	1.53 (0.66, 4.24)	0.005 **	8.39 ± 7.64	7.04 (1.78, 12.51)	0.014 *	0.002 **	0.012 *

Abbreviations: SD, standard deviation; IQR, interquartile range; S–W test, Shapiro–Wilk test; BMI, body mass index; KL grade, Kellgren–Lawrence grade; PA, physical activity; LPA, light physical activity; MVPA, moderate-to-vigorous physical activity. ^#^ S–W test: *p* < 0.05 indicates that the data are not assumed to be normally distributed. * *p* < 0.05, ** *p* < 0.01.

**Table 2:** Logistic regression model of factors correlated with undergoing TKA.

Independent Variable	β	Odds Ratio	*p*-Value	95% CI
KL grade	1.38	3.97	0.049 *	1.01–15.66
Knee pain	1.01	2.75	0.019 *	1.18–6.42

β: partial regression coefficient. Abbreviations: KL grade, Kellgren-Lawrence grade. Independent variables (KL grade, knee pain, steps, and MVPA) were fed in by the stepwise method for dependent variables. * *p* < 0.05.

## Data Availability

The data used to this study are available from the corresponding author upon request.
